# Role of Neoadjuvant Chemotherapy in Non-small Cell Lung Cancer in the COVID-19 Pandemic

**DOI:** 10.7759/cureus.29720

**Published:** 2022-09-28

**Authors:** Elanur Karaman, Arife Ulas, Arif Hakan Onder, Adem Deligonul, Sibel O Orhan, Atilla Pekcolaklar

**Affiliations:** 1 Medical Oncology, Medical Park Karadeniz Hospital, Trabzon, TUR; 2 Medical Oncology, University of Health Sciences, Bursa City Education and Research Hospital, Bursa, TUR; 3 Medical Oncology, Antalya Education and Research Hospital, Antalya, TUR; 4 Medical Oncology, Uludag University School of Medicine, Bursa, TUR; 5 Medical Oncology, Uludag University Faculty of Medicine, Bursa, TUR; 6 Thoracic Surgery, University of Health Sciences, Bursa City Education and Research Hospital, Bursa, TUR

**Keywords:** objective response rate, pathological complete response, non-small cell lung cancer (nsclc), covid-19, neo-adjuvant chemotherapy

## Abstract

Introduction

Cancer patients are among the groups at high risk in the COVID-19 pandemic. Here, we aimed to determine the effectiveness of neoadjuvant chemotherapy (NACT) during the pandemic period and examine the prognostic factors in patients with non-small cell lung cancer (NSCLC).

Method

Patients with stage I-III NSCLC were treated in our hospitals between 2020-2022. Treatment responses were evaluated in patients who underwent NACT. Prognostic factors and the nutritional and inflammatory indexes were investigated.

Results

Thirty-eight patients received NACT. 57.9% of patients were stage-III. The objective response rate was 57.9%. Pathological complete response was obtained in 10.5% of patients. No prognostic role of inflammatory indices was determined. 21.1% of patients developed a COVID-19 infection. Disease-free survival was 19 months. Survival decreased with large tumor size and presence of metastasis.

Conclusion

NACT has high response rates. NACT can be used as bridging therapy in suitable patients whose surgery is postponed during the pandemic period.

## Introduction

Since December 2019, the world has been struggling with coronavirus disease 2019 (COVID-19) [1]. It has been reported that approximately 13% of cancer patients diagnosed with COVID-19 have severe respiratory symptoms, and 2% die. Most of the deaths in the COVID-19 epidemic were in patients over 60 years of age and with comorbidities [2].

Lung cancer is the second most common and deadliest cancer in men [3]. Lung cancer patients are at high risk in the COVID-19 pandemic. During the active period of the pandemic, elective surgeries were postponed due to health policies. The presence of tumor downgrading with neoadjuvant chemotherapy (NACT) may offer the possibility of surgery in inoperable cases and lobectomy in cases that require pneumonectomy [4,5].

Oncological treatments were applied in malignant patients in line with the recommendations of health policies or guidelines and by taking the necessary precautions regarding the pandemic. This situation has brought the application of bridging treatments such as chemotherapy and radiotherapy to patients whose surgery was postponed [6].

Our primary aim was to determine the efficacy of NACT in non-metastatic non-small cell lung cancers (NSCLCs) during the COVID-19 pandemic period. Our second aim was to evaluate the prognostic factors. Finally, our third aim was to increase clinicians' awareness of the effects of the COVID-19 pandemic on cancer patients.

## Materials and methods

Patients with resectable or potentially resectable NSCLCs who applied to the medical oncology outpatient clinic treated between 2020 and 2022 were evaluated for inclusion in the study.

The study was carried out in accordance with the Declaration of Helsinki of 1975, as revised in 2000. Data usage permission was obtained from the relevant institutions, and Ethics Committee approval was obtained.

Inclusion and exclusion criteria

Patients who were 18-80 years old, had NACT therapy in the first line with the diagnosis of resectable or potentially resectable NSCLC, had surgery for NSCLC after NACT, and had no COVID-19 infection before treatment were included in the study. Having metastases, having another cancer, having a disease that causes immunosuppression or using drugs/substances, and having an active infection at the beginning of treatment were all part of the exclusion criteria.

Patients' demographic and clinical features, COVID-19 transmission status, tumor histology, tumor pathological features (grade, lymph node status, postoperative stage, type of operation, lymphovascular invasion, perineural invasion, and surgical margin), and administered chemotherapy drugs were examined.

American Joint Committee on Cancer eighth edition was used for tumor, node, metastases (TNM) classification in clinical and pathological staging [7]. Changes were noted before and after treatment by imaging and pathology reports. Treatment responses (complete response (CR), partial response (PR), stable disease (SD), and progressive disease) were evaluated with Response Evaluation Criteria in Solid Tumors (RECIST) criteria [8]. Disease-free survival (DFS) was calculated as the time of diagnosis to recurrence, and overall survival (OS) was calculated as the time of diagnosis to death or last follow-up.

Neutrophil lymphocyte ratio (NLR), platelet lymphocyte ratio (PLR), monocyte lymphocyte ratio (MLR), hemoglobin albumin lymphocyte and platelet (HALP) score (hemoglobin (g/L)×albumin (g/L)×lymphocytes (/L)/platelets (/L)) were examined [9-11]. The presence of anemia was evaluated as hemoglobin <120g/L, and hypoalbuminemia was determined as albumine <40g/L. Area under the curve (ROC) analysis was performed to determine the threshold level of NLR, PLR, MLR and HALP scores. No results were obtained for NLR, PLR, MLR, and HALP score in the ROC curve; median values were used in the evaluation.

Treatment Methods

A multidisciplinary team decided on the operation of the patients who received NACT. NACT regimens were three to four cycles of gemcitabine 1000-1250mg/m2 + cisplatin 75mg/m2 or carboplatin AUC(5), docetaxel 75mg/m2 + cisplatin 75mg/m2 or carboplatin AUC(5), paclitaxel 175mg/m2 + carboplatin AUC(5-6) or cisplatin 75mg/m2, and vinorelbine 25-30mg/m2 + cisplatin 75mg/m2 [12-15].

A preoperative pulmonary function test was performed. It was aimed to perform complete anatomic resection in patients who underwent NACT. If the tumor invades the pulmonary fissure or main pulmonary artery, pneumonectomy was performed regardless of the tumor site and the type of NACT. On the other hand, lobectomy was performed in other situations. Wedge resection or segmentectomy was preferred for patients with respiratory reserve problems. Moreover, systematic mediastinal/hilar lymph node dissection was always performed in all patients.

Patients receiving neo-adjuvant immunotherapy were not included in the study due to limited access and the risk of cytokine storm [16].

Patients were followed up every three months for two-three years with physical examination and laboratory techniques. Contrast-enhanced chest tomography was performed one month after NACT. Subsequently, they were followed up with thorax tomography every six months.

Statistics

After the obtained data were coded with numerical values, they were analyzed with SPSS version 20 (IBM Inc., Armonk, New York). Complementary statistics of the evaluation results were given as numbers and percentages for categorical variables and as median, standard deviation, minimum, and maximum for numerical variables. The Kaplan-Meier method was used to obtain the progression-free survival curve. Factors affecting survival were evaluated with Cox regression analysis. Confidence interval was determined as 95%, and a p-value <0.05 for statistical significance.

## Results

A total of 38 patients were included in the study; 33 patients (86.8%) were male and five (13.2%) were female. Demographic, clinical, and tumor characteristics of patients are shown in Table 1.

**Table 1:** Demographic, clinical, and tumor characteristics of patients ECOG: Eastern Cooperative Oncology Group, PS: performance status

Characteristics of patients		
Age, mean		61.5±7.6
Gender, n (%)	Male	33 (86.8)
	Female	5 (13.2)
Comorbidities, n (%)	Yes	28 (73.7)
	No	10 (26.3)
Tumor size (cm), median		5.1 (3.2-7)
Smoking (pack/year), median		40 (30-50)
ECOGperformance status, n (%)	ECOG PS 0-1	37 (97.4)
	ECOG PS 2-3	1 (2.6)
Body mass index, n (%)	<18.5-24.9	16 (42.1)
	≥ 25	22 (57.9)
Histology, n (%)	Adenocarcinoma	16 (42.1)
	Squamous cell carcinoma	22 (57.9)
Clinical stage, n (%)	Stage I A, B	4 (10.5)
	Stage II A	1 (2.6)
	Stage II B	11 (28.9)
	Stage III A	12 (31.6)
	Stage III B	9 (23.7)
	Stage III C	1 (2.6)
Getting COVID infection, n (%)	Yes	8 (21.1)
	No	30 (78.9)
Treatment completed, n (%)	Yes	36 (97.4)
	No	2 (2.6)
Stage after neoadjuvant chemotherapy	Stage 0	4 (10.5)
	Stage I A, B	6 (15.8)
	Stage IIA	3 (7.9)
	Stage II B	12 (31.6)
	Stage III A	12 (31.6)
	Stage IIIB	1 (2.6)
	Stage IIIC	-
Response, n (%)	Complete response	4 (10.5)
	Partial response	18 (47.4)
	Stable disease	13 (34.2)
	Progressive disease	3 (7.9)
Recurrence or metastases, n (%)	Present	13 (34.2)
	Absent	25 (65.8)

While nine patients (23.7%) were T1, six patients (15.8%) were T2, 13 patients (34.2%) were T3, and 10 patients (26.3%) were T4; 22 patients (57.9%) were N0-1, and 16 patients (42.1%) were N2-3. When the clinical stages of patients were examined, 22 (57.9%) were found to be stage-III. A COVID-19 infection was detected in eight (21.1%) patients.

After NACT, 30 (78.9) patients underwent lobectomy, four (10.5%) pneumonectomy, two (5.3%) segmentectomy, and two (5.3%) lobectomy and segmentectomy/wedge dissection operations. The median number of lymph nodes removed was 18.5 (12.75-29). R1 (resection margin) was detected in four (10.5%) patients. Visceral pleural invasion was observed in 36.8% (14) patients, lymphovascular invasion in 50% (19) patients, and perineural invasion in 6.3% (1/16) patients. COVID-19 infection was detected in eight (21.1%) patients. No intensive care hospitalization or mortality associated with COVID-19 infection was observed. Thirty-six patients (97.4%) of patients were able to complete their treatment. 

Fluorodeoxyglucose (FDG)-positron emission tomography (PET)/CT was performed for staging purposes in 37 patients (97.3%). After the NACT administration, the mean maximum standardized uptake value (SUVmax) values of the primary tumor and lymph nodes were decreased (p<0.001, p<0.001).

During a median follow-up of 12 (7-18) months, 36 patients (97.4%) were able to complete their therapies. Pathologic complete response (PCR) was obtained in four (4/38 10.5%) patients, and partial response was obtained in 18 (47.4%) patients (Table 1). When the stages after the treatments were examined, it was seen that 25 patients (65.8%) were stage-III, three patients (7.9%) were stage-II, and 10 patients (26.3%) were stage-I or below. Postoperatively, N0 was detected in 16 (42.1%) patients, N1 in 15 (39.5%) patients, N1+2 in six (15.8%) patients, and N2 in one (2.6%) patient. Tumor reduction was seen in 30 (78.9%) patients, lymph node reduction in 16 (53.3%) patients, and downstage in 22 (57.9%) patients who received NACT. Progressive disease was detected in three patients (7.9%, Table 2).

**Table 2:** Factors affecting overall survival in univariate Cox regression analysis CR: complete response, ECOG: Eastern Cooperative Oncology Group, SUVmax: maximum standardized uptake value, HALP: hemoglobin albumin lymphocyte and platelet

Variables	Univariate Analysis HR (95% CI)	p-value	Multivariate analysis HR (95% CI)	p-value
Age (years) ≥65/<65	1.162 (0.259-5.210)	0.844		
Gender male/female	0.496 (0.096-2.569)	0.404		
Response status, CR/others	0.039 (0.000-531.814)	0.505		
Objective response rates, Yes/No	1.775 (0.344-9.155)	0.493		
ECOG performance status, ≥2/0–1	6.795 (0.756-61.058)	0.087		
Comorbidities presence/absence	33.212 (0.029-38026.224)	0.330		
Tumor size	1.357 (1.029-1.790)	0.031	0.670 (0.068-6.582)	0.731
Getting COVID infection Yes/No	1.888 (0.364-9.780)	0.449		
Local recurrence presence/absence	2.383 (0.530-10.725)	0.258		
Metastasis presence/absence	5.733 (1.107-29.695)	0.037	6.295 (1.091-36.316)	0.040
Smoking, pack/year	1.026 (0.991-1.062)	0.142		
Body mass index ≥25/<25	0.654 (0.146-2.938)	0.580		
Treatment before SUVmax value	0.993 (0.885-1.114)	0.903		
Presence of anemia presence/absence	2.331 (0.451-12.056)	0.313		
Presence of hypoalbuminemia presence/absence	3.611 (0.807-16.154)	0.093		
Presence of thrombocytosis presence/absence	1.445 (0.280-7.472)	0.660		
Neutrophil lymphocyte ratio ≥1.476/<1.476	1.816 (0.404-8.150)	0.436		
Platelet lymphocyte ratio ≥0.10/<0.10	1.370 (0.305-6.168)	0.681		
Monocyte lymphocyte ratio ≥26.42<26.42	3.870 (0.745-20.090)	0.107		
HALP score ≥7.5/<7.5	1.695 (0.326-8.812)	0.530		
Presence of N2 after treatment presence/absence	4.124 (0.901-18.882)	0.068		
Stage reduction after treatment presence/absence	1.014 (0.227-4.537)	0.985		
Post-treatment stage-III presence/absence	2.913 (0.644-13.180)	0.165		
Pre-treatment stage-III presence/absence	54.106 (0.111-26458.072)	0.207		

Objective response rates (CR+PR) were 57.9%. Four patients with PCR were treated with a platinum- and taxane-based NACT regimen. Response rates and objective response rates are similar in NACT regimens (p=0.052, p=0.081). In taxane and platinum-based regimens, no correlation was found between the taxane (paclitaxel vs. docetaxel) or platinum (cisplatin vs. carboplatin) type and response rates (p=0.390, p=0.955). Furthermore, there was no statistical difference found in primary tumor size reduction rates, stage reduction status, and lymph node reduction status after the operation between the NACT regimens (p=0.831, 0.081, and 0.288, respectively).

There was no difference between tumor histology with primary tumor size reduction rate, lymph node reduction status, stage reduction status, response rates, and objective response rate (ORR) (p=1.000, 1.000, 0.612, 0.347, and 0.612, respectively). However, all PCRs were achieved in patients with squamous cell carcinoma.

Anemia was found in 50% of patients, thrombocytosis in 18.4%, and hypoalbuminemia in 28.9%. Recurrence was detected in seven patients (18.4%), and metastasis was detected in nine patients (23.7%). The median DFS was 19 months (95% CI NA). The two-year survival rate was 74%.

In the univariate Cox regression analysis, a large tumor size and the presence of metastasis reduced survival (p=0.031, p=0.037, Table 2). In the multivariate Cox regression analysis, the presence of metastasis negatively affected survival (p=0.040, Table 2). Neither inflammatory indices, nor COVID-19 infection nor response to treatment affected survival.

Among 38 patients who underwent tumor resection, 10 (26.3%) patients did not receive any adjuvant chemotherapy or radiotherapy. Thirty (78.9%) patients received adjuvant chemotherapy of the same or different drug according to treatment response. Four (10.5%) patients received adjuvant radiotherapy for R1 resection margin, and two (5.3%) patients received radiotherapy for extranodal extension of the residual metastatic mediastinal lymph node.

## Discussion

NACT is a bridging treatment option in operable NSCLCs during the pandemic period. In our study, PCR rate was 10.5% with NACT. NACT treatment resulted in significant objective response rates and increased survival. FDG-PET/CT SUVmax values decreased with oncological treatments. About one-fifth of patients had COVID-19 infection during NACT.

Lung cancer is the most common cause of cancer-related death in both men and women [3]. About 30% of NSCLC is detected in the early stage, and the main treatment for which cure is achieved is surgery [17,18]. Neoadjuvant treatments show the response of the tumor to treatment. Early initiation of systemic therapy allows micrometastases to be controlled. In addition, patients have higher compliance rates and performance status before surgery [19]. Furthermore, meta-analyses demonstrated the survival benefit of NACT in NSCLC [20,21]. In our study, it was determined that the survival rate was increased in patients who underwent NACT treatment protocol.

The application of active oncological treatments to patients during the pandemic period increases the risk of COVID-19 infection. As in our study, most NSCLC patients are elderly, immunocompromised, with excessive comorbidities, and are among the patients at high risk of being affected by the pandemic. During the NACT treatment process, which was applied in line with the recommendations of local health policies during the pandemic period [2].

Chemotherapy and immunotherapy can be used in neoadjuvant treatment of NSCLCs.Tyrosine kinase inhibitors are only effective in patients with target mutations, but their neoadjuvant use has not yet been approved. Our study was conducted during the active period of the COVID-19 pandemic, and the use of immunotherapies was limited due to the possibility of cytokine release syndrome during the pandemic period and especially the pulmonary side effects that could develop [16]. In addition, a phase 3 study related to the use of neoadjuvant immunotherapy was published in May 2022, when the pandemic began to lose its effect [22].

It is known in the literature that squamous cell cancers (SCCs) respond better to NACT than adenocancers and PCR rates vary 0-34% [23,24]. In our study, the PCR rate among those who received NACT was consistent with literature, and all patients with PCR had SCC histology.

High FDG-PET/CT SUVmax levels in the primary tumor are a poor prognostic factor in NSCLC. In imaging performed to evaluate the response to oncological treatments, changes in tumor metabolism will predict prognosis [25]. In our study, a significant decrease was observed in FDG-PET/CT SUVmax levels with NACT treatments, but the effect of this situation on prognosis could not be evaluated due to the small number of patients.

COVID-19 can affect the host's immune system [26]. Due to the limited number of patients in our study, inflammatory scores did not affect survival. There are conflicting results in the literature regarding the prognostic role of inflammatory indices. In a study conducted by Winther-Larsen et al. [27], high NLR, PLR, and MLR were found to be associated with decreased OS, but no correlation was found between HALP score and survival in 5320 stage I-IV non-small cell lung cancer patients. It is thought that the difference in the analyzed patient stages, the effects of oncological treatments, and genetic factors may cause this difference in the literature. More comprehensive studies are needed to evaluate the effect of inflammatory indices on prognosis in these patients.

The limitations of our study are the retrospective nature and limited patient group. Patients receiving neoadjuvant or adjuvant immunotherapy could not be evaluated.

## Conclusions

NACT has high response rates, although one-fifth of patients were infected with Covid 19 during the Covid-19 pandemic. Higher PCR is observed in SCCs. Primary tumor and lymph node FDG-PET CT SUVmax levels measured before and after treatment may predict response to NACT. When necessary precautions are provided, NACT can be considered as a bridge treatment during the pandemic period for resectable or potentially resectable NSCLC patients. Prospective studies with a larger number of patients are needed to evaluate the effect of inflammatory and nutritional indices on prognosis in this patient group.

## References

[1] Bogoch II, Watts A, Thomas-Bachli A, Huber C, Kraemer MU, Khan K (2020). Pneumonia of unknown aetiology in Wuhan, China: potential for international spread via commercial air travel. J Travel Med.

[2] Liang W, Guan W, Chen R (2020). Cancer patients in SARS-CoV-2 infection: a nationwide analysis in China. Lancet Oncol.

[3] Siegel RL, Miller KD, Jemal A (2019). Cancer statistics, 2019. CA Cancer J Clin.

[4] Kumar S, Saikia J, Kumar V Jr, Malik PS, Madan K, Jain D, Bharati S (2020). Neoadjuvant chemotherapy followed by surgery in lung cancer: Indian scenario. Curr Probl Cancer.

[5] Weissferdt A, Pataer A, Vaporciyan AA (2020). Agreement on major pathological response in NSCLC patients receiving neoadjuvant chemotherapy. Clin Lung Cancer.

[6] Kidane B, Spicer J, Kim JO, Fiset PO, Abdulkarim B, Malthaner R, Palma D (2020). SABR-BRIDGE: Stereotactic ABlative Radiotherapy Before Resection to AvoId Delay for Early-Stage LunG Cancer or OligomEts during the COVID-19 pandemic. Front Oncol.

[7] Rami-Porta R, Asamura H, Travis WD, Rusch VW (2017). Lung cancer - major changes in the American Joint Committee on Cancer eighth edition cancer staging manual. CA Cancer J Clin.

[8] Eisenhauer EA, Therasse P, Bogaerts J (2009). New response evaluation criteria in solid tumours: revised RECIST guideline (version 1.1). Eur J Cancer.

[9] Yuan C, Li N, Mao X, Liu Z, Ou W, Wang SY (2017). Elevated pretreatment neutrophil/white blood cell ratio and monocyte/lymphocyte ratio predict poor survival in patients with curatively resected non-small cell lung cancer: results from a large cohort. Thorac Cancer.

[10] Ding N, Pang Z, Shen H, Ni Y, Du J, Liu Q (2016). The prognostic value of PLR in lung cancer, a meta-analysis based on results from a large consecutive cohort. Sci Rep.

[11] Zhai B, Chen J, Wu J (2021). Predictive value of the hemoglobin, albumin, lymphocyte, and platelet (HALP) score and lymphocyte-to-monocyte ratio (LMR) in patients with non-small cell lung cancer after radical lung cancer surgery. Ann Transl Med.

[12] Van Kooten M, Rosenberg M, Orlando M (2002). Neoadjuvant chemotherapy with gemcitabine and cisplatin in stage IIIA/B non-small cell lung cancer. Invest New Drugs.

[13] Liao WY, Chen JH, Wu M (2013). Neoadjuvant chemotherapy with docetaxel-cisplatin in patients with stage III N2 non-small-cell lung cancer. Clin Lung Cancer.

[14] O'Brien ME, Splinter T, Smit EF (2003). Carboplatin and paclitaxol (Taxol) as an induction regimen for patients with biopsy-proven stage IIIA N2 non-small cell lung cancer. an EORTC phase II study (EORTC 08958). Eur J Cancer.

[15] Palka M, Sanchez A, Córdoba M (2017). Cisplatin plus vinorelbine as induction treatment in stage IIIA non-small cell lung cancer. Oncol Lett.

[16] Tay SH, Toh MM, Thian YL (2022). Cytokine release syndrome in cancer patients receiving immune checkpoint inhibitors: a case series of 25 patients and review of the literature. Front Immunol.

[17] Groome PA, Bolejack V, Crowley JJ, Kennedy C, Krasnik M, Sobin LH, Goldstraw P (2007). The IASLC Lung Cancer Staging Project: validation of the proposals for revision of the T, N, and M descriptors and consequent stage groupings in the forthcoming (seventh) edition of the TNM classification of malignant tumours. J Thorac Oncol.

[18] Gaur P, Bhattacharya S, Kant S, Kushwaha RA, Singh G, Pandey S (2018). EGFR mutation detection and its association with clinicopathological characters of lung cancer patients. World J Oncol.

[19] Sun L, Guo YJ, Song J (2020). Neoadjuvant EGFR-TKI therapy for EGFR-mutant NSCLC: a systematic review and pooled analysis of five prospective clinical trials. Front Oncol.

[20] NSCLC Meta-analysis Collaborative Group (2014). Preoperative chemotherapy for non-small-cell lung cancer: a systematic review and meta-analysis of individual participant data. Lancet.

[21] Song WA, Zhou NK, Wang W (2010). Survival benefit of neoadjuvant chemotherapy in non-small cell lung cancer: an updated meta-analysis of 13 randomized control trials. J Thorac Oncol.

[22] Forde PM, Spicer J, Lu S (2022). Neoadjuvant nivolumab plus chemotherapy in resectable lung cancer. N Engl J Med.

[23] Akyıl M, Tezel Ç, Tokgöz Akyıl F (2020). Prognostic significance of pathological complete response in non-small cell lung cancer following neoadjuvant treatment. Turk Gogus Kalp Damar Cerrahisi Derg.

[24] Qu Y, Emoto K, Eguchi T (2019). Pathologic assessment after neoadjuvant chemotherapy for NSCLC: importance and implications of distinguishing adenocarcinoma from squamous cell carcinoma. J Thorac Oncol.

[25] Kim N, Kim JS, Geol Lee C (2020). Predictive value of interim 18F-FDG-PET in patients with non-small cell lung cancer treated with definitive radiation therapy. PLoS One.

[26] Wool GD, Miller JL (2021). The impact of COVID-19 disease on platelets and coagulation. Pathobiology.

[27] Winther-Larsen A, Aggerholm-Pedersen N, Sandfeld-Paulsen B (2022). Inflammation-scores as prognostic markers of overall survival in lung cancer: a register-based study of 6,210 Danish lung cancer patients. BMC Cancer.

